# Solvent-Free Reaction for Unsymmetrical Organodisulfides with High Purity and Application as Cathode-Active Materials

**DOI:** 10.3390/ma17030699

**Published:** 2024-02-01

**Authors:** Yuta Tsukaguchi, Kazuki Shinoda, Yusei Noda, Yui Hatta, Kentaro Tsubouchi, Naoko Shokura, Fumiya Nakamura, Hiromi Kimura-Suda, Hirofumi Yoshikawa, Takeshi Shimizu, Naoki Tanifuji

**Affiliations:** 1 Chemistry and Biochemistry Division, Department of Integrated Engineering, National Institute of Technology, Yonago College, 4448 Hikona-cho, Yonago 683-8502, Japanc19431@yonago-k.ac.jp (Y.N.);; 2 Chitose Institute of Science and Technology, 758-65 Bibi, Chitose 066-8655, Japan; 3 Department of Material Science, School of Engineering, Kwansei Gakuin University, Gakuen 2-1, Sanda 669-1337, Japan

**Keywords:** disulfide bond, unsymmetrical disulfide, disproportionation, solvent-free reaction, rechargeable battery

## Abstract

Unsymmetrical disulfides, in which different organic groups are bonded to disulfide bonds, have been synthesized by cross-coupling reactions using thiols as substrates. However, due to the low-binding energy of unsymmetrical disulfides, its disproportionation occurs based on the side reactions with nucleophilic thiols, resulting in the impurity of symmetric disulfides. In this study, we developed a solvent-free synthesis method for unsymmetrical disulfides using thiosulfonates, thiols, and a base. This synthetic method enabled us to obtain highly pure diaryl-substituted unsymmetrical disulfides with particularly low-binding energy without control over the nucleophilicity and elimination properties of the substrate. Furthermore, it was observed that the disproportionation of unsymmetrical disulfides occurred in the solvent. This means that solvent-free condition is one of the factors to obtain unsymmetrical disulfides. As a new application of unsymmetrical disulfides, we applied unsymmetrical disulfides to cathode active materials of lithium batteries based on the reversible multi-electron redox activity of S–S bonds. The batteries using unsymmetrical disulfide cathode-active materials with a carbon nanotube exhibited initial capacities of 127 and 158 Ah/kg, equal to 42 and 53% of their theoretical ones. We demonstrated that unsymmetrical disulfides could be used as cathode-active materials for rechargeable batteries.

## 1. Introduction

A disulfide (S–S) bond is a covalent bond composed of two sulfur atoms, which is formed through the thiol oxidation of cysteine residues in vivo. Organic compounds with S–S bonds are essential substructures that maintain the three-dimensional structure of proteins and functional peptides in organisms [1] and have recently been used as electrochemical functional materials, such as cathode-active materials of rechargeable batteries [2] and biosensors [3], based on the redox activity of S–S bonds. In particular, unsymmetrical disulfides, which have different organic groups at both ends of the S–S bond, not only exhibit important properties as pharmaceuticals, such as suppressing Pseudomonas aeruginosa infection [4] and anticancer activity [5] but also as materials for self-assembled monolayers [6]. As described above, unsymmetrical disulfide compounds have been attractive research subjects from the viewpoint of their application as functional materials, the elucidation of their functions, and the development of synthetic methods.

**Scheme 1:**
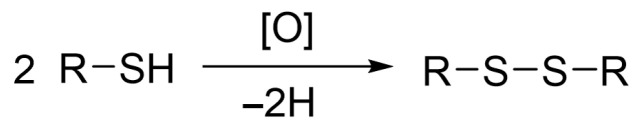
Synthesis of symmetric disulfide.

**Scheme 2:**
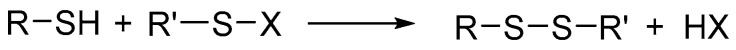
Synthesis of unsymmetrical disulfide.

According to previous reports, unsymmetrical disulfides, unlike symmetric disulfides, have been synthesized using thiols and sulfides [7,8]. As shown in Scheme 1, symmetric disulfides can be easily synthesized by oxidizing one type of thiol in a solution [9]. In the case of using two types of disulfides based on this reaction, resulting in not only an unsymmetrical disulfide but also two symmetric disulfides as byproducts, have been synthesized because of oxidation reactions between the same thiols [7,8]. Therefore, to precisely synthesize unsymmetrical disulfides, as shown in Scheme 2, the method has been used in which a thiol (R-SH) nucleophilically attacks a sulfide (R′-S-X) consisting of leaving group X and a different hydrocarbon group R′. However, unsymmetrical disulfides have suffered from disproportionation because cleavage/recombination of S–S bonds at room temperature has produced stable symmetric disulfides as a by-product [7,10,11,12,13]. This disproportionation has been triggered by the lower bond energy of the S–S bond (20–26 kcal/mol) of diaryl disulfides than that of the peroxide (O–O) bond (32 kcal/mol) [14,15]. Therefore, in the course of the coupling reaction, thiols have reacted with disulfides as nucleophiles, making it impossible to obtain highly pure unsymmetrical disulfides in the solution, where molecular collisions occur frequently because of disproportionation based on the exchange reaction between S–S, and S–H bonds [7,8]. In this work, we developed a synthetic method for highly pure unsymmetrical disulfides under solvent-free conditions, where molecular motions are restricted. Also, we applied unsymmetrical disulfides as the cathode-active materials of rechargeable batteries to utilize the electrochemical multi-electron redox reaction of S–S bonds [2,16]. Recently, organic compounds have been reported as lightweight cathode-active materials with higher theoretical capacity densities than conventional metal oxide-based materials [17,18].

## 2. Materials and Methods

### 2.1. Materials

All materials were used without further purification. Diphenyl disulfide, di-*p*-tolyl disulfide, 4,4′-dichlorodiphenyl disulfide, 4,4′-dibromodiphenyl disulfide, sodium benzenesulfinate dihydrate, *p*-toluenethiol, 4-cholorobenzenethiol, 4-bromobenzenethiol, 4-choloroaniline, and *p*-toluenesulfonic acid monohydrate were purchased from Tokyo Chemical Incorporation (Tokyo, Japan). Diphenyl disulfide and iodine were obtained from FUJIFILM Wako Pure Chemical Corporation (Osaka, Japan).

### 2.2. Synthesis

**Scheme 3:**
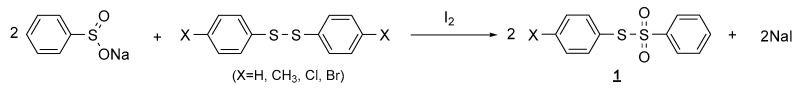
Synthesis of thiosulfonate **1**.

**Scheme 4:**
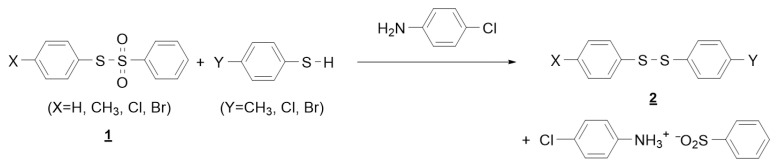
Synthesis of unsymmetrical disulfide **2**.

The unsymmetrical disulfide compounds were obtained via two-step synthesis. First, as shown in Scheme 3, diphenyl disulfide **1**, an intermediate of the unsymmetrical disulfide compound, was obtained via a method. Second, as shown in Scheme 4, unsymmetrical disulfide compound **2** was obtained via solvent-free synthesis using **1**.

#### 2.2.1. **1a** (X = H)

The synthesis of **1a** was followed by Scheme 3. First, diphenyl disulfide (0.437 g, 2.00 mmol) was added to the CH_2_Cl_2_ solution of sodium benzenesulfinate dihydrate (0.985 g, 6.00 mmol) in a 20 mL sample tube and suspended by a shaking apparatus (AS ONE, Osaka, Japan, ASONE TRIO TM-1) for 1 min. After adding iodine (1.02 g, 4.00 mmol), the suspension was shaken for 1 min again and then heated in an aluminum block thermostatic chamber (Taitec, Tokyo, Japan, DTU-1C) at 30 °C for 24 h. Second, the resultant solid was extracted by 50 mL of dichloromethane and separated after adding 5% sodium thiosulfate as the after-treatment of unreacted iodine. The organic layer, separated twice with saturated brine, was dried using magnesium sulfate. After filtering off the magnesium sulfate, the oily product was obtained via evaporation at 30 °C. Finally, the oily product was purified using column chromatography. In total, 5 mL of silica gel, which absorbed the oily product, was charged on the top of the column (diameter: 20 mm) and then developed with n-hexane to remove the reactant. **1a** (yield: 0.484 g) was obtained as a colorless solid after development with and the evaporation of dichloromethane.

^1^H NMR (400 MHz, CDCl_3_) δ = 7.30–7.50 (m, 7H), 7.56–7.62 (m, 3H); 13C NMR (101 MHz, CDCl_3_) δ = 127.6, 127.9, 128.8, 129.46, 131.43, 133.6, 136.6, 143.0.

#### 2.2.2. **1b** (X = CH_3_)

**1b** was synthesized by the same process as that of **1a** but using di-*p*-tolyl disulfide (0.493 g, 2.00 mmol) instead of diphenyl disulfide.

^1^H NMR (400 MHz, CDCl_3_) δ = 2.38 (s, 3H), 7.11–7.17 (m, 2H), 7.20–7.28 (m, 2H), 7.40–7.48 (m, 2H), 7.54–7.63 (m, 2H); ^13^C NMR (101 MHz, CDCl_3_) δ = 21.5, 124.4, 127.6, 128.8, 130.2, 133.5, 133.5, 142.2, 143.2.

#### 2.2.3. **1c** (X = Cl)

**1c** was synthesized by the same process as that for **1a** but using 4,4′-dichlorodiphenyl disulfide (0.574 g, 2.00 mmol) instead of diphenyl disulfide.

^1^H NMR (400 MHz, CDCl_3_) δ = 7.14–7.28 (m, 4H), 7.34–7.43 (m, 2H), 7.47–7.54 (m, 3H); ^13^C NMR (101 MHz, CDCl_3_) δ = 126.4, 127.6, 129.0, 129.8, 133.88, 137.7, 138.3, 142.8.

#### 2.2.4. **1d** (X = Br)

**1d** was synthesized by the same process as that of **1a** but using 4,4′-dibromodiphenyl disulfide (0.752 g, 2.00 mmol) instead of diphenyl disulfide.

^1^H NMR (400 MHz, CDCl_3_) δ = 7.16–7.28 (m, 2H), 7.42–7.52 (m, 2H), 7.54–7.62 (m, 3H); ^13^C NMR (101 MHz, CDCl_3_) δ = 126.7, 127.0, 127.6, 129.0, 132.8, 133.9, 137.9, 142.9.

#### 2.2.5. **2a** (X = H, Y = CH_3_)

The synthesis of **2a** was followed by Scheme 4. The mixture of **1a** (0.748 g, 3.00 mmol) and *p*-toluenethiol (0.248 g, 2.00 mmol) was ground homogeneously in an agate mortar. The solvent-free reaction was finished by adding 4-choloroaniline (0.382 g, 3.00 mmol) and grinding for 1 min. Moreover, to deal with excess 4-choloroaniline, the mixture was ground again after adding the *p*-toluenesulfonic acid monohydrate (0.190 g, 1.00 mmol). Next, the mixture was extracted with 25 mL of *n*-hexane, and the product was separated through short column chromatography (diameter: 2.5 cm, length: 1.5 cm). **2a** (yield: 0.381 g, 82%) was obtained as a colorless solid after the evaporation of *n*-hexane.

^1^H NMR (400 MHz, CDCl_3_) δ = 2.31 (s, 3H), 7.05–7.13 (m, 2H), 7.17–7.42 (m, 5H), 7.45–7.53 (m, 2H); ^13^C NMR (101 MHz, CDCl_3_) δ = 21.04, 127.06, 127.61, 128.34, 129.00, 129.82, 133.60, 137.28, 137.49; MS (EI): *m*/*z* (%) = 232 (M+, 100), 168 (13), 187 (35), 123 (69), 109 (14).

#### 2.2.6. **2b** (X = H, Y = Cl)

**2b** was synthesized by the same process as that of **2a** but using 4-chlorobenzenethiol (0.289 g, 2.00 mmol) instead of *p*-toluenethiol.

^1^H NMR (400 MHz, CDCl_3_) δ = 7.20–7.34 (m, 5H), 7.37–7.51 (m, 4H); ^13^C NMR (101 MHz, CDCl_3_) δ = 127.45, 127.80, 129.04, 129.15, 129.19, 133.28, 135.62, 136.56; MS (EI): *m*/*z* (%) = 252 (M+, 100), 188 (23), 187 (35), 143 (45), 109 (71).

#### 2.2.7. **2c** (X = H, Y = Br)

**2c** was synthesized by the same process as that of **2a** but using 4-bromobenzenethiol (0.378 g, 2.00 mmol) instead of *p*-toluenethiol.

^1^H NMR (400 MHz, CDCl_3_) δ = 7.04–7.13 (m, 2H), 7.17–7.33 (m, 3H), 7.34–7.41 (m, 2H), 7.45–7.52 (m, 2H); ^13^C NMR (101 MHz, CDCl_3_) δ = 21.04, 127.06, 127.61, 128.34, 129.00, 129.82, 133.60, 137.28, 137.49; MS (EI): *m*/*z* (%) = 232 (M+, 100), 168 (13), 187 (35), 123 (69), 109 (14).

#### 2.2.8. **2d** (X = Cl, Y = CH_3_)

**2d** was synthesized by the same process as that of **2a** but using **1b** (0.790 g, 3.00 mmol) and 4-cholorobenzenethiol (0.289 g, 2.00 mmol) instead of **1a** and *p*-toluenethiol.

^1^H NMR (400 MHz, CDCl_3_): δ = 2.32 (3H, s), 7.24–7.29 (2H, m), 7.31–7.36 (2H, m), 7.37–7.45 (4H, m); ^13^C NMR (101 MHz, CDCl_3_): δ = 21.06, 128.71, 129.14, 129.20, 129.92, 133.18, 133.21, 135.90, 137.88; MS (EI): *m*/*z* (%) = 266 (M+, 71), 123 (100), 108 (17).

#### 2.2.9. **2e** (X = Br, Y = Cl)

**2e** was synthesized by the same process as that of **2a** but using **1c** (0.851 g, 3.00 mmol) and 4-bromobenzenethiol (0.378 g, 2.00 mmol) instead of **1a** and *p*-toluenethiol.

^1^H NMR (400 MHz, CDCl_3_) δ = 7.24–7.30 (m, 2H), 7.31–7.36 (m, 2H), 7.37–7.45 (m, 4H); ^13^C NMR (101 MHz, CDCl_3_) δ = 121.57, 129.34, 129.47, 132.25, 133.69, 135.11, 135.83; MS (EI): *m*/*z* (%) = 322 ([M + 2]+, 100), 320 (M+, 72), 251 (16), 187 (35), 143 (46), 108 (87).

#### 2.2.10. **2f** (X = Br, Y = CH_3_)

**2f** was synthesized by the same process as that of **2a** but using **1b** (0.790 g, 3.00 mmol) and 4-bromobenzenethiol (0.378 g, 2.00 mmol) instead of **1a** and *p*-toluenethiol.

^1^H NMR (400 MHz, CDCl_3_) δ = 2.35 (s, 3H), 7.06–7.13 (m, 2H), 7.33–7.47 (m, 6H); ^13^C NMR (101 MHz, CDCl_3_) δ = 121.12, 128.69, 129.33, 129.97, 132.08, 133.12, 136.58, 137.93; MS (EI): *m*/*z* (%) = 310 (M+, 76), 231 (12), 123 (100), 108 (23).

#### 2.2.11. 1,4-di(Phenyldithio)benzene **2g**

**Scheme 5:**
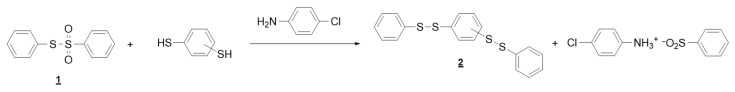
Synthesis of di(phenyldithio)benzene.

The synthesis of di(phenyldithio)benzene was followed by Scheme 5. The mixture of **1a** (0.875 g, 3.50 mmol) and 1,4-benzenedithiol (0.142 g, 1.00 mmol) was ground homogeneously in an agate mortar. The solvent-free reaction was performed by adding 4-choloroaniline (0.214 g, 2.00 mmol) and grinding for one min. Moreover, to deal with excess 4-choloroaniline, the mixture was ground again after adding the *p*-toluenesulfonic acid monohydrate (0.190 g, 1.00 mmol). Next, the mixture was extracted with 25 mL of *n*-hexane, and the product was separated through short column chromatography (diameter: 2.5 cm, length: 1.5 cm). 1,4-di(phenyldithio)benzene (yield: 0.318 g, 82%) was obtained as a pale yellow solid after the evaporation of *n*-hexane.

^1^H NMR (400 MHz, CDCl_3_) δ = 7.20–7.32 (m, 6H), 7.41 (s, 4H), 7.45–7.49 (m, 4H); ^13^C NMR (101 MHz, CDCl_3_) δ = 127.4, 127.6, 128.2, 129.1, 136.3, 136.7

#### 2.2.12. 1,3-di(Phenyldithio)benzene **2h**

1,3-di(Phenyldithio)benzene was synthesized by the same process as that of 1,4-di(phenyldithio)benzene, but using 1,3-benzenedithiol (0.142 g, 1.00 mmol) instead of 1,4-benzenedithiol.

^1^H NMR (400 MHz, CDCl_3_) δ = 7.17–7.33 (m, 9H), 7.40–7.45 (m, 4H), 7.69 (t, 1H, J = 1.4 Hz); ^13^C NMR (101 MHz, CDCl_3_) δ = 124.9, 125.5, 127.3, 127.7, 129.1, 129.5, 136.5, 138.4.

### 2.3. Observation of Disproportionation Reaction of Unsymmetrical Disulfide Compounds

The disproportionation reaction of the unsymmetrical disulfide compound was observed via the gradual change in the ^13^C NMR chart of an unsymmetrical disulfide at 25 °C. To observe the disproportionation clearly from the change in the number of carbons, **2d** was chosen as the target material. First, the ^13^C NMR measurement of **2d** dissolved in CDCl_3_ was performed immediately. Second, the same measurements were performed after 4, 16, and 32 days, respectively. In comparison, the above measurements were performed using symmetric disulfide compounds bis(4-methylphenyl) disulfide and bis(4-chlorophenyl) disulfide synthesized using the same method as that of **2a**.

### 2.4. Evaluation of Battery Performances

#### 2.4.1. Battery Fabrication

The thin film cathode was fabricated as follows. First, unsymmetrical organodisulfides were added into carbon black (Toka black 5500, Tokai carbon, Aichi, Japan), and it was ground in an agate mortar for 30 min. After that, the polyvinylidene difluoride (PVDF) binder (Kishida chemicals, Osaka, Japan) was added and ground again for 1 h. The weight ratio of unsymmetrical organodisulfide, carbon black, and PVDF was 30:50:20. The mixture was stirred in *N*-methylpyrrolidone. The prepared slurry was coated evenly onto aluminum foil (thickness: 20 μm) using a doctor blade technique and was dried at room temperature under a vacuum overnight at room temperature. The foil was cut into a disc with a diameter φ of 15.95 mm. The active material loading was ca. 0.405 mg/cm^2^. CR2032 coin-type cells were assembled using the cathode, lithium foil (φ: 15.50 mm) as an anode, including the polypropylene film separator, and 1 M LiClO_4_ in TEGDME in an Ar-filled glovebox.

#### 2.4.2. Discharge–Charge Measurements

Galvanostatic discharge/charge tests (voltage: 1.5–3.5 V, current density: 1000 mA g^−1^) were performed at room temperature (25 °C) on a Hokuto Denko HJ1020mSD8 charge/discharge device. The rate performances of **2g** and **2h** were evaluated at rates of 100, 200, 500, and 1000 mA g^−1^ on a charge/discharge device (Hokuto Denko, Himonya, Japan, HJ1020mSD8).

#### 2.4.3. Electrochemical Impedance Spectroscopy (EIS) Measurements

EIS measurements of **2g** and **2h** were performed at room temperature (25 °C) at rest voltages of the batteries, including **2g** and **2h** (3.1 V) on HZ-Pro S12 (Hokuto Denko, Himonya, Japan).

#### 2.4.4. Cyclic Voltammetry (CV) Measurements

CV measurements of **2g** and **2h** were carried out at room temperature (25 °C) at a scanning rate of 0.2 mV s^−1^ between 1.5 and 3.0 V on HZ-Pro S12 (Hokuto Denko).

## 3. Results and Discussion

The purities of thiosulfonates and unsymmetrical disulfides were judged from the results of NMR, MS, and Ramman data. According to the ^1^H and ^13^C NMR spectra (Figures S1–S8), each thiosulfonate with high purity was obtained only via liquid separation and short-column treatment. Also, Raman spectra of **2a**, **2b**, **2c**, **2d**, **2e**, and **2f** (Figure 1a,b) showed the peaks assigned to S–S bond vibrations between 450 and 550 cm^−1^ [19], indicating that the S–S bonds were formed via the solvent-free method. It was confirmed that the unsymmetrical disulfides with high purity were obtained from the NMR, MS, and IR spectra (Figures S9–S24). Furthermore, as shown in Table 1, Table 2 and Table 3, four types of thiosulfonate salt and six unsymmetrical disulfides were obtained with a yield of over 85% and 80%, respectively. Surprisingly, this solvent-free method realized the high yield of unsymmetrical disulfides [5,7,8].

**Scheme 6:**
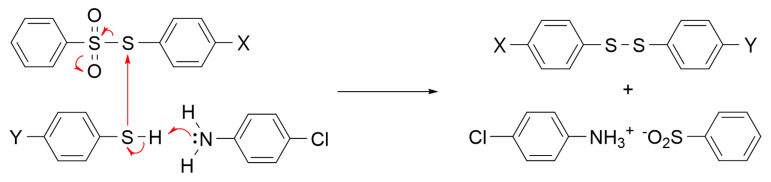
The reaction mechanism of the solvent-free method for unsymmetrical disulfides.

This solvent-free reaction was expected to proceed through a two-step mechanism (Scheme 6). Note that the reaction did not proceed with just adding thiol to sulfonate. The reaction proceeded rapidly when aniline in appropriately equimolar amounts to the thiol was added to the mixture. This is because the protonation of aniline is a driving force. The protons of the thiol were polarized by the lone pair of aniline, resulting in a nucleophilic attack from the divalent sulfur of the thiol on the divalent sulfur in sulfonate, an electron-withdrawing sulfonyl group [20], and the elimination of the sulfonyl group as a good leaving group. In the case of previous solvent reactions, thiols also reacted with the unsymmetrical disulfide product, resulting in symmetric disulfides as by-products based on S–S and S–H exchange reactions. However, in the case of solvent-free conditions, this side reaction did not occur, maintaining the purity and yield of the unsymmetrical disulfides. Furthermore, two improvements made it possible to increase the purity of the unsymmetrical disulfide and promote the solvent-free reaction. (Improvement 1) An excessive amount of sulfonate was determined; this improvement could completely consume the thiol, preventing it from reacting with the unsymmetrical disulfide during solvent extraction. The excessive amount of sulfonate could be easily removed via short chromatography and then reused. (Improvement 2) Excessive aniline was observed; excess aniline activated all thiols and became a cation that paired with sulfinic acid (R-SO^2−^), promoting the reaction. Excess aniline was completely removed via salt formation with *p*-toluenesulfonic acid, solvent extraction, and chromatography.

**Figure 2:**
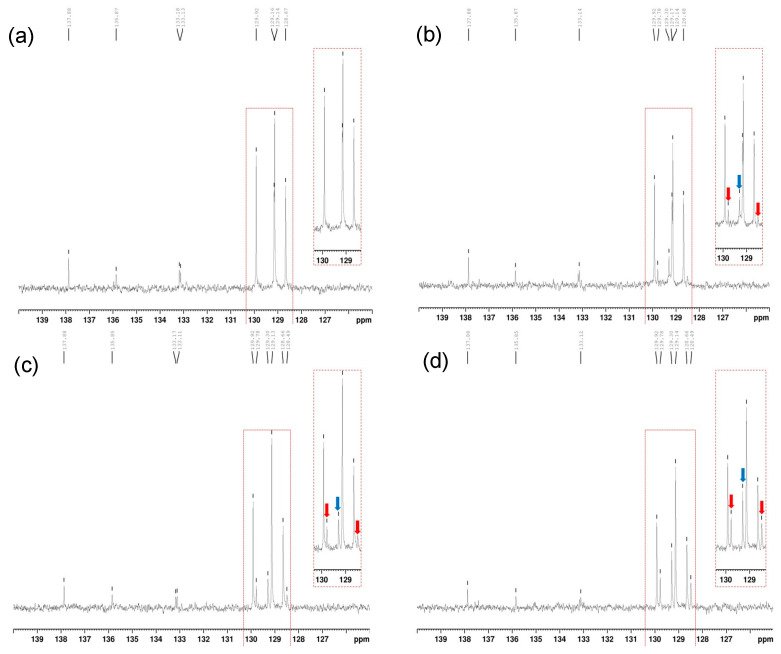
The change in ^13^C NMR spectra of **2d** after 0 (**a**), 4 (**b**), 16 (**c**), and 32 days (**d**) (bis(4-methylphenyl) disulfide (red arrow) and bis(4-chlorophenyl) disulfide (blue arrow)).

**Figure 3:**
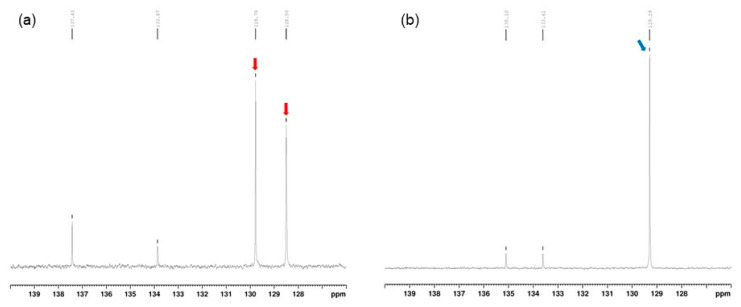
^13^C NMR spectra of bis(4-methylphenyl) disulfide (**a**) and bis(4-chlorophenyl) disulfide (**b**).

**Scheme 7:**
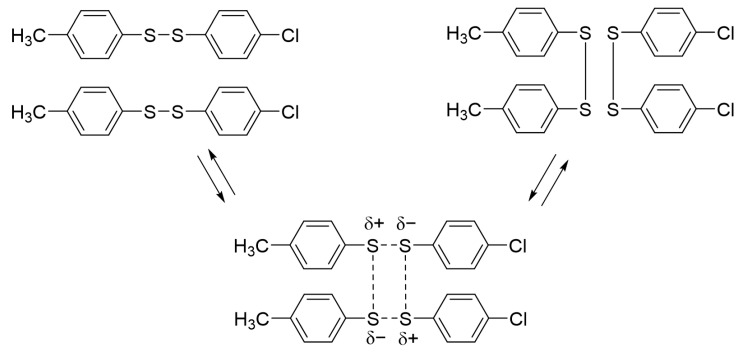
S–S exchange reaction and disproportionation of unsymmetrical disulfide.

**Scheme 8:**
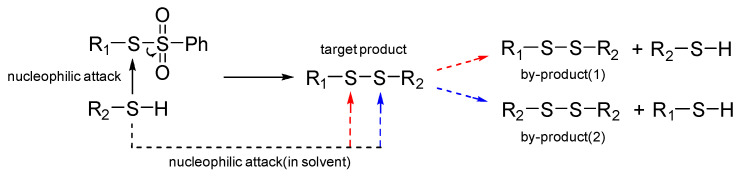
The disproportionation mechanism of unsymmetrical disulfide in the course of the reaction. The red and blue arrows indicate the reactions of by-product (1) and (2), respectively.

To evaluate the stability of unsymmetrical diaryl disulfide in the solution, we tracked the ^13^C NMR spectral changes of **2d** dissolved in chloroform-d. Figure 2a shows that three peaks were observed around 128–130 ppm in the ^13^C NMR spectrum immediately after solution preparation. However, new peaks with low intensities appeared at around 128–130 ppm in the ^13^C-NMR spectrum after four days (Figure 2b), and after 16 days (Figure 2c,d), the intensities of the peaks assigned to bis(4-methylphenyl) disulfide (δ = 128.50, 129.78 ppm; Figure 3a) and bis(4-chlorophenyl) disulfide (δ = 129.29 ppm; Figure 3b) were enhanced. These results indicate that the unsymmetrical disulfide was transformed into two types of symmetric disulfides through self-disproportionation due to the S–S exchange reaction in the solution state at room temperature (Scheme 7). However, the bond cleavage was not very fast because not only were the peaks assigned to two types of symmetric disulfides, but also those of unsymmetrical disulfides were observed. This suggests that the nucleophilic attack from the thiol reagent to the disulfide was the main cause of disproportionation (Scheme 8). We believe that the solvent-free condition suppressed the above-mentioned nucleophilic attack, making it possible to obtain a highly pure unsymmetrical disulfide.

**Figure 4:**
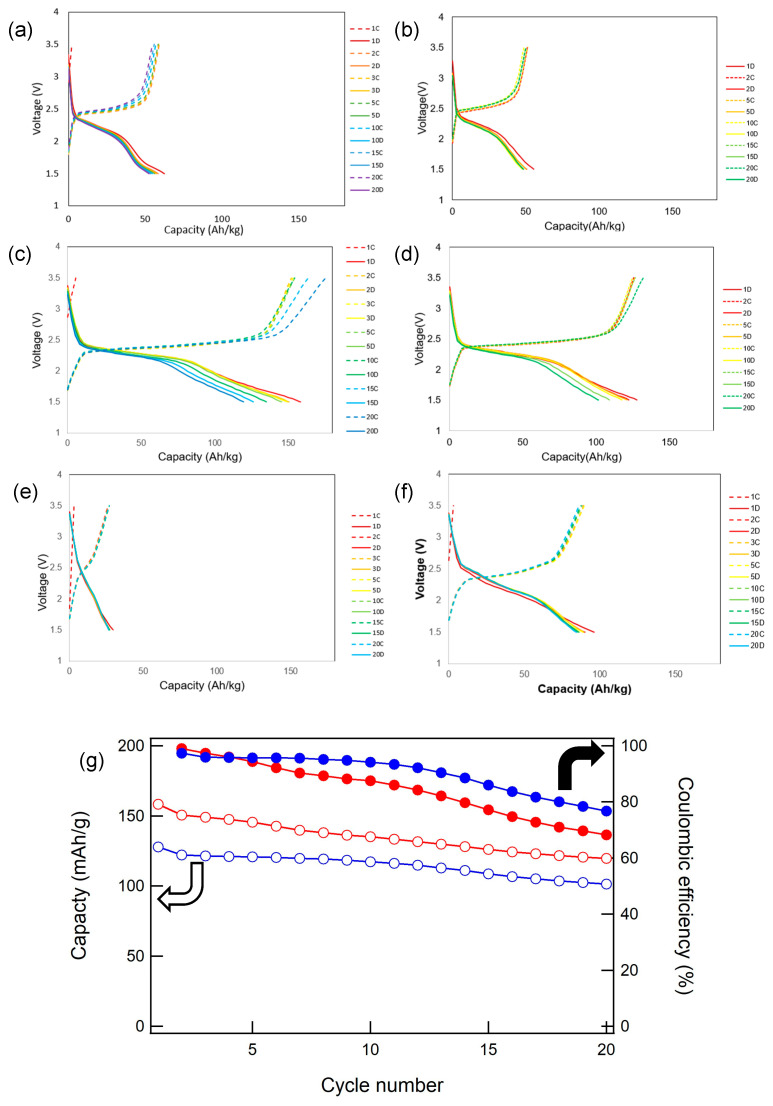
Discharge–charge curves of unsymmetrical disulfides at **2g** (**a**), **2h** (**b**), **2g** with CNT (**c**), and **2h** with CNT (**d**). Discharge–charge curves of diphenyl disulfide without CNT (**e**) and with CB/CNT (**f**). (**g**) Cycle performance of **2g** (red) and **2h** (blue) with CNT.

Figure 4a–d show the charge–discharge curves of a lithium rechargeable battery using unsymmetrical disulfides **2g** and **2h** (theoretical capacity: 299 Ah/kg) as cathode-active materials. The plateaus were observed in the charge and discharge curves at around 2.5 V [16], corresponding to the redox peaks of S–S bonds of **2g** and **2h** in cyclic voltammograms (Figure S25). In addition, **2g** and **2h** exhibited the initial capacities of 62 (Figure 4a) and 55 Ah/kg (Figure 4b), and the capacity retentions of 84 and 87% were identified after their initial ones for 20 cycles, respectively. These indicate that **2g** and **2h** showed electrochemical reactions based on the cleavage/recombination of S–S bonds, although their capacities were equal to only 18–21% of their theoretical ones. In other words, only 18–21% of the S–S bonds reacted due to the poor electronic conductivities of common organosulfids. According to previous research, lithium thiolate anions, which are very soluble in the electrolyte, were generated after discharge, resulting in low-capacity retention, but **2g** and **2h** exhibited relatively higher capacity retention because only a small amount of **2g** and **2h** reacted. On the other hand, when 10% of the conductive carbon was replaced with CNT, **2g** and **2h** exhibited capacities of 158 (Figure 4c) and 127 Ah/kg (Figure 4d), equal to 53 and 42% of their theoretical values, respectively. This suggests that the π–π interaction between the CNT and the benzene ring in **2g** and **2h** enabled electrons to transfer from the conductive additive CNT surface to **2g** and **2h**. In addition, diphenyl disulfide (theoretical capacity: 245 Ah/kg) mixed with only CB exhibited an initial capacity of 30 Ah/kg (Figure 4e), equal to 12% of the theoretical values. On the other hand, diphenyl disulfide mixed with CB and CNT exhibited a capacity of 96 Ah/kg (Figure 4f), equal to 39% of the theoretical value. These suggest that the S–S bonds with two different substituents induced a stronger polarization than those with the same substituents and resulted in more efficient electrochemical reactions [21]. Furthermore, the stabilities of **2g** and **2h** with CNT were evaluated. First, as shown in Figure 4g, the capacity (**2g** with CNT: 120–158 mAh/g, **2h** with CNT: 101–127 mAh/g) and Coulombic efficiencies (**2g**: 68–99%, **2h**: 77–97% with CNT) gradually dropped with every cycle. Second, their rate performances became worse as cycle numbers increased (Figure S26) in spite of their lower charge transfer resistance than the organodisulfides previously reported (Figure S27) [22,23]. We think that the worse cycle and rate performances were caused by the instabilities of **2g** and **2h**, such as the low bond energy of the S–S bond in organodisulfides with two aryl groups (20–26 kcal/mol) and the solubility of the liquid electrolyte after the discharge process.

## 4. Conclusions

We successfully synthesized unsymmetrical disulfides ay good yields without disproportionation under solvent-free conditions. In particular, it was worth synthesizing unsymmetrical diaryl disulfides with high purities. It was revealed that the thiol as a substrate did not nucleophillically attack the S–S bonds in the target product under solvent-free conditions. Furthermore, unsymmetrical diaryl disulfides exhibited good electrochemical performances as the cathode-active materials of rechargeable batteries. We believe that unsymmetrical diaryl disulfides have the potential as the cathode-active materials of next-generation batteries because of their intermolecular polarization and the π–π interaction between carbon materials and diaryl moieties in the future.

## Figures and Tables

**Figure 1:**
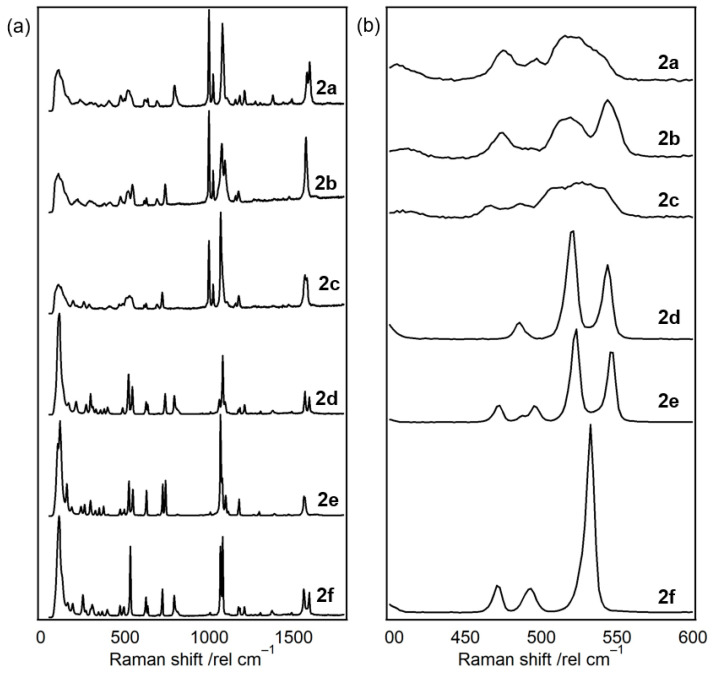
Raman spectra of unsymmetrical disulfides **2a**, **2b**, **2c**, **2d**, **2e**, and **2f** in the range of 0–1800 (**a**) and 400–600 cm^−1^ (**b**).

**Table 1:** Thiosulfonates synthesized in this work.

R in R-S-S-R	Product	Yield [%]
phenyl	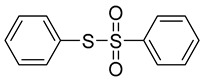 **1a**	97
4-methylphenyl	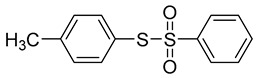 **1b**	85
4-chlorophenyl	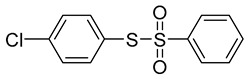 **1c**	89
4-bromophenyl	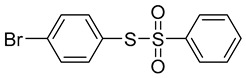 **1d**	89

**Table 2:** Unsymmetrical disulfides synthesized in this work.

R in R-S-SO_2_-Ph	R′ in R′-SH	Product (R-SS-R′)	Yield [%]
phenyl	4-methylphenyl	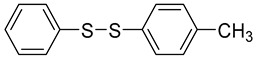 **2a**	82
phenyl	4-chlorophenyl	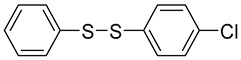 **2b**	96
phenyl	4-bromophenyl	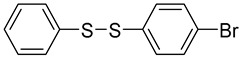 **2c**	99
4-chlorophenyl	4-methylphenyl	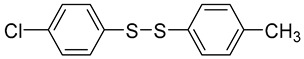 **2d**	87
4-chlorophenyl	4-bromophenyl	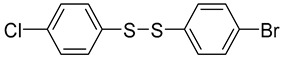 **2e**	87
4-bromophenyl	4-methylphenyl	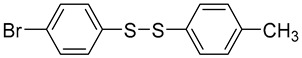 **2f**	89

**Table 3:** Di(phenyldithio)benzene synthesized in this work.

R in R-S-SO_2_-Ph	R′ in R′-SH	Product (R-SS-R′)	Yield [%]
phenyl	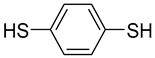	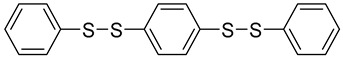 **2g**	75
phenyl	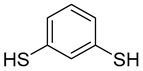	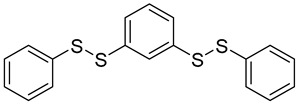 **2h**	96

## Data Availability

Data are contained within the article.

## Supplementary Materials

The following supporting information can be downloaded at: https://www.mdpi.com/article/10.3390/ma17030699/s1. Figures S1–S8: ^1^H and ^13^C NMR spectra of thiosulfonates; Figures S9–S24: ^1^H and ^13^C NMR spectra of unsymmetrical disulfides; Figure S25: Cyclic voltammograms of **2g** and **2h**; Figure S26: Rate performances of **2g** and **2h**; Figure S27: Nyquist plot of **2g** and **2h**. Table S1: Charge transfer resistances of organodisulfides.
