# Supplementary material for: Solvent-Free Reaction for Unsymmetrical Organodisulfides with High Purity and Application as Cathode-Active Materials
Source: Materials (Basel). 2024 Feb 1;17(3):699. doi: 10.3390/ma17030699 (PMC10856588; doi:10.3390/ma17030699)
Supplement: Supplementary file 1 [file materials-17-00699-s001.zip › materials-2774487-supplementary.pdf]

materials

MDPI

Supplementary Materials

Solvent-Free Reaction for Unsymmetrical Organodisulfides with High Purity and Application as Cathode Active Materials

Yuta Tsukaguchi, Kentaro Tsubouchi, Kazuki Shinoda, Yui Hatta, Yusei Noda, Fumiya Nakamura, Hiromi Kimura-Suda, Hirofumi Yoshikawa, Takeshi Shimizu, Naoki Tanifiuji*

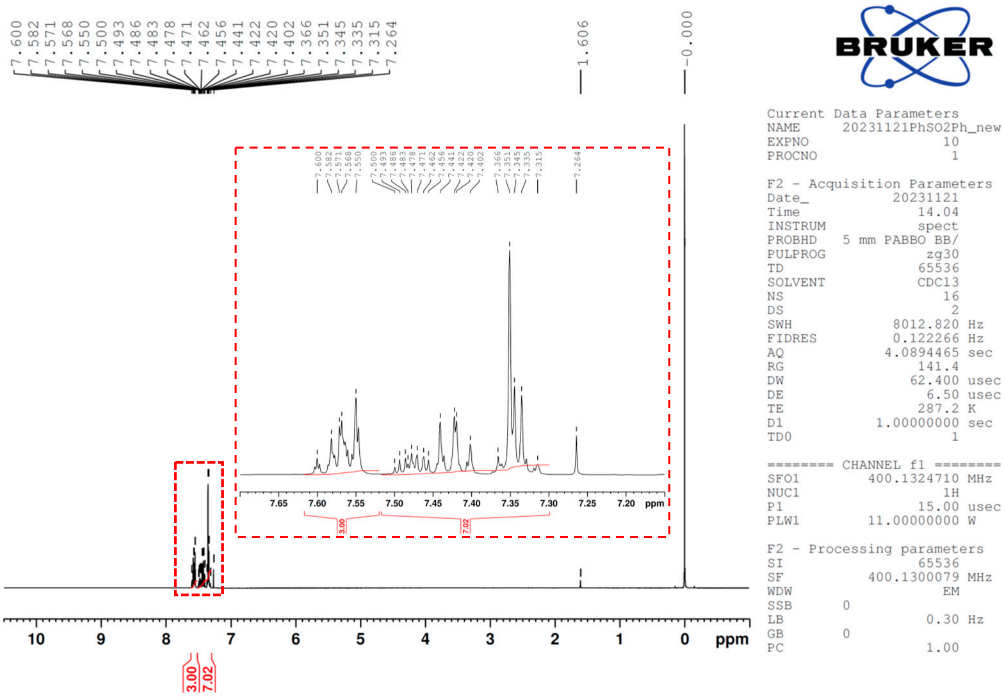

Figure S1. ^{1}H NMR spectrum of of **1a**

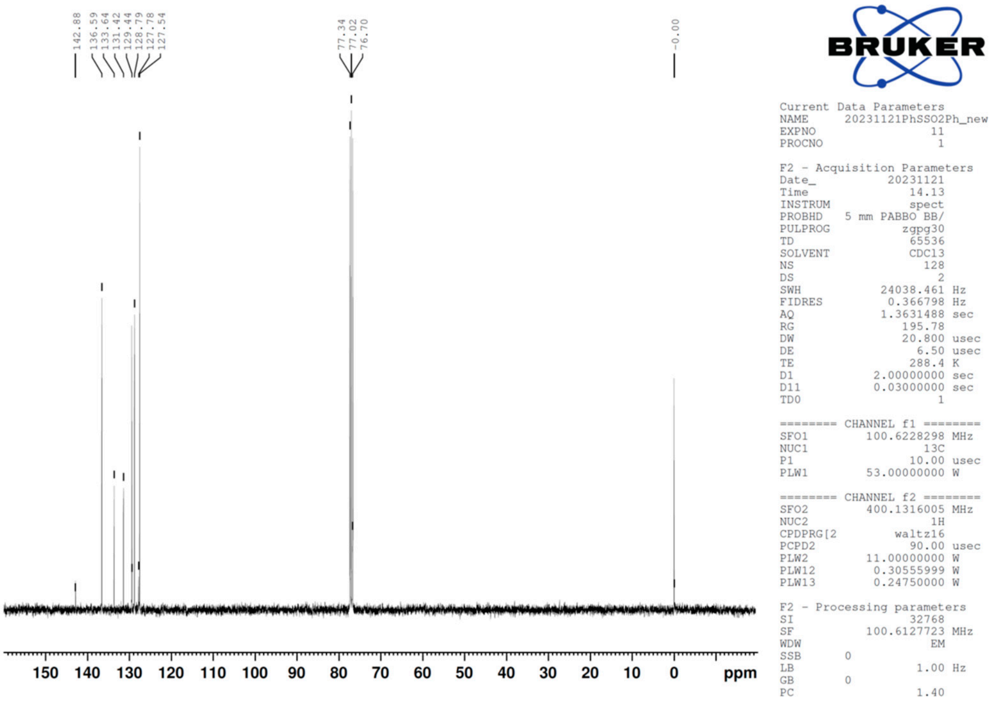

Figure S2. ^{13}C NMR spectrum of **1a**

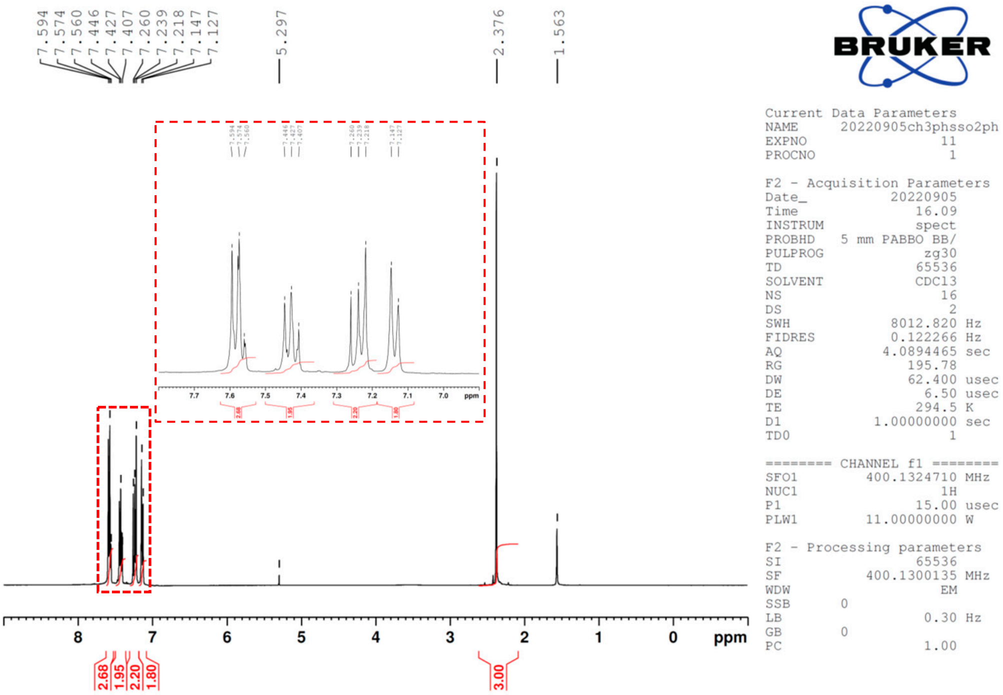

Figure S3. ^{1}H NMR spectrum of **1b**

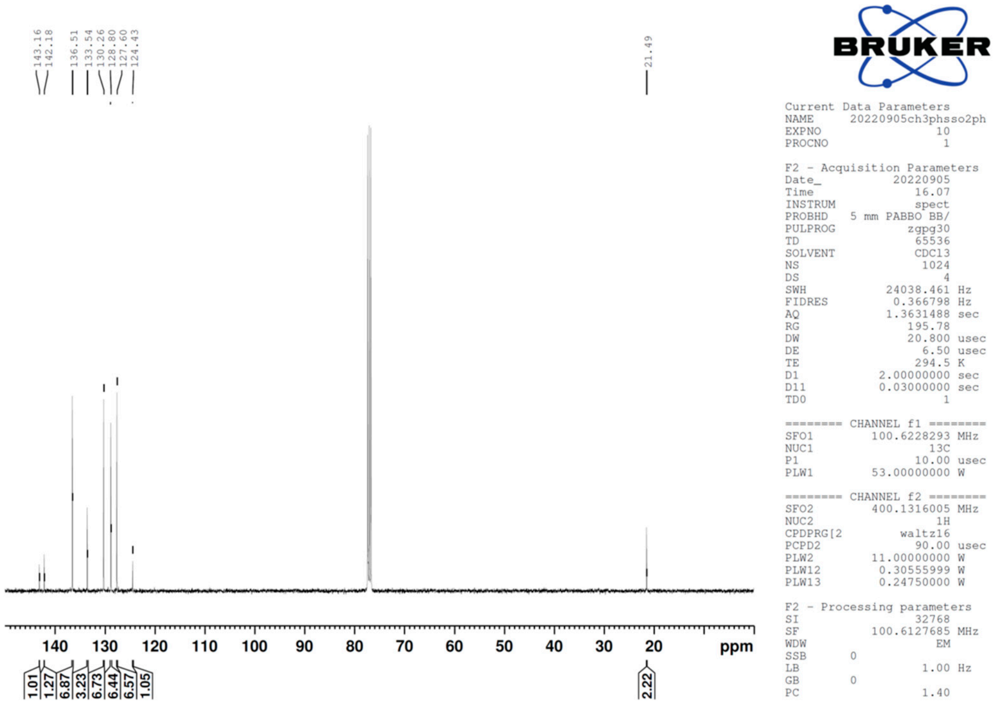

Figure S4. ^{13}C NMR spectrum of **1b**

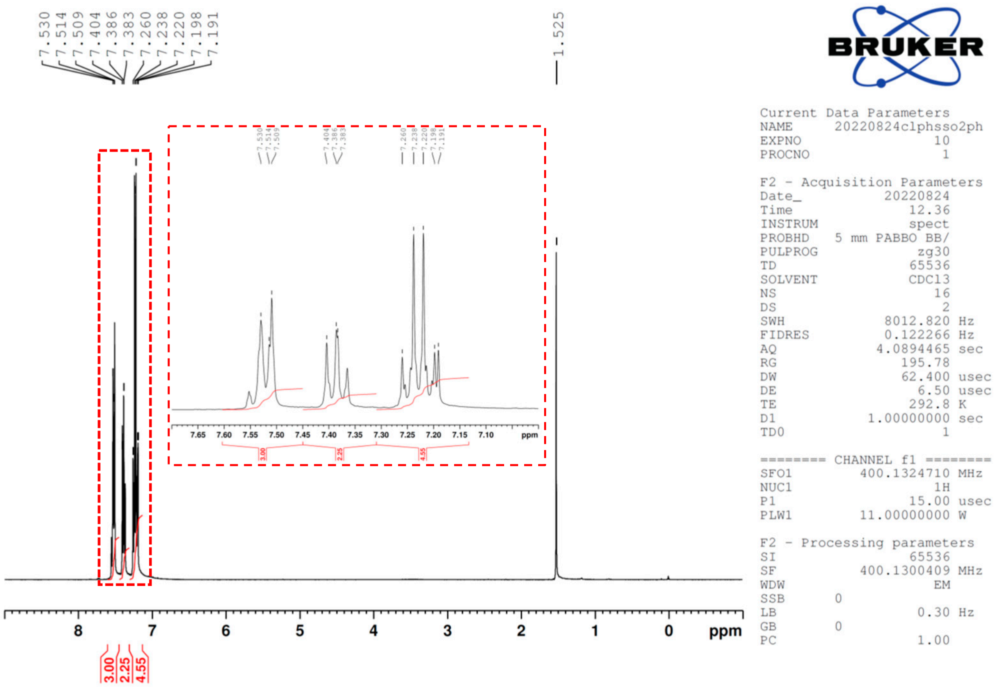

Figure S5. ^{1}H NMR spectrum of **1c**

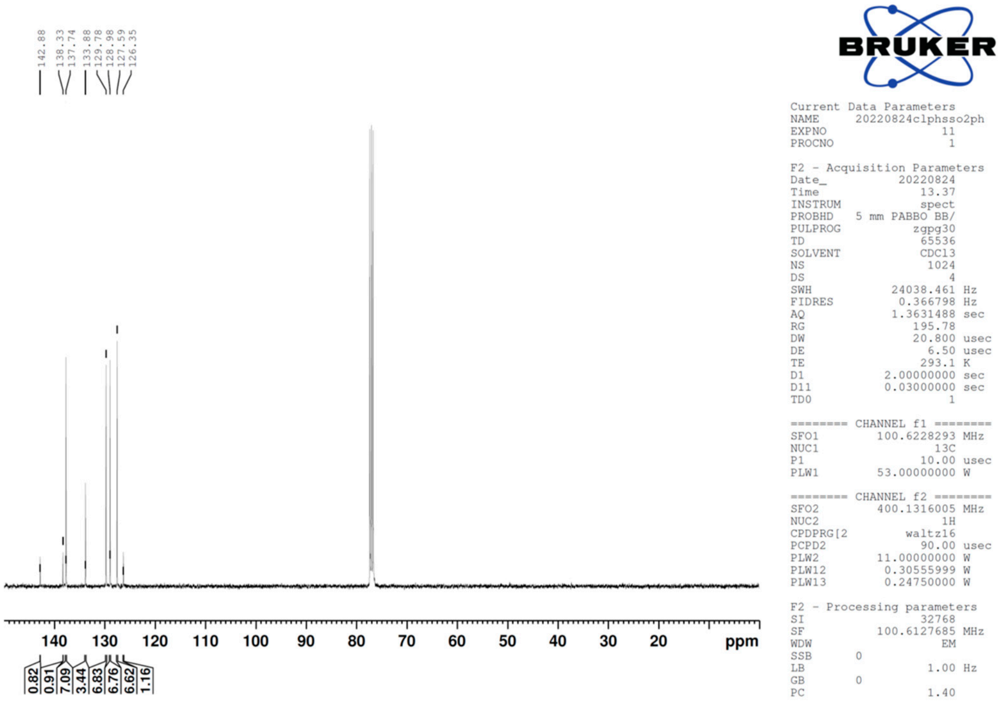

Figure S6. ^{13}C NMR spectrum of **1c**

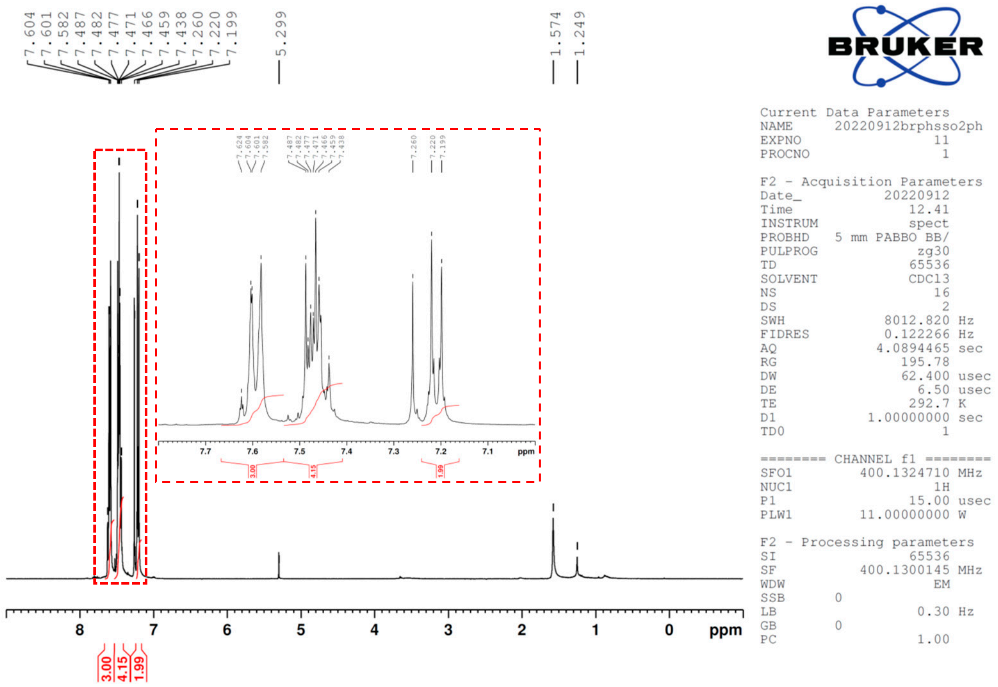

Figure S7. ^{1}H NMR spectrum of **1d**

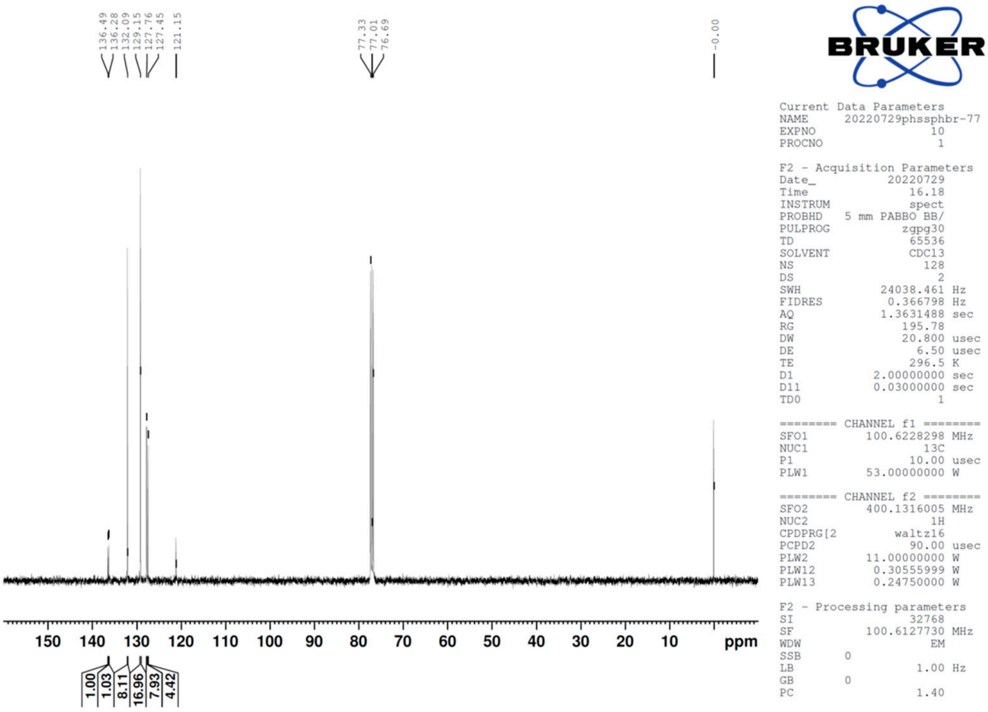

Figure S8. ^{13}C NMR spectrum of **1d**

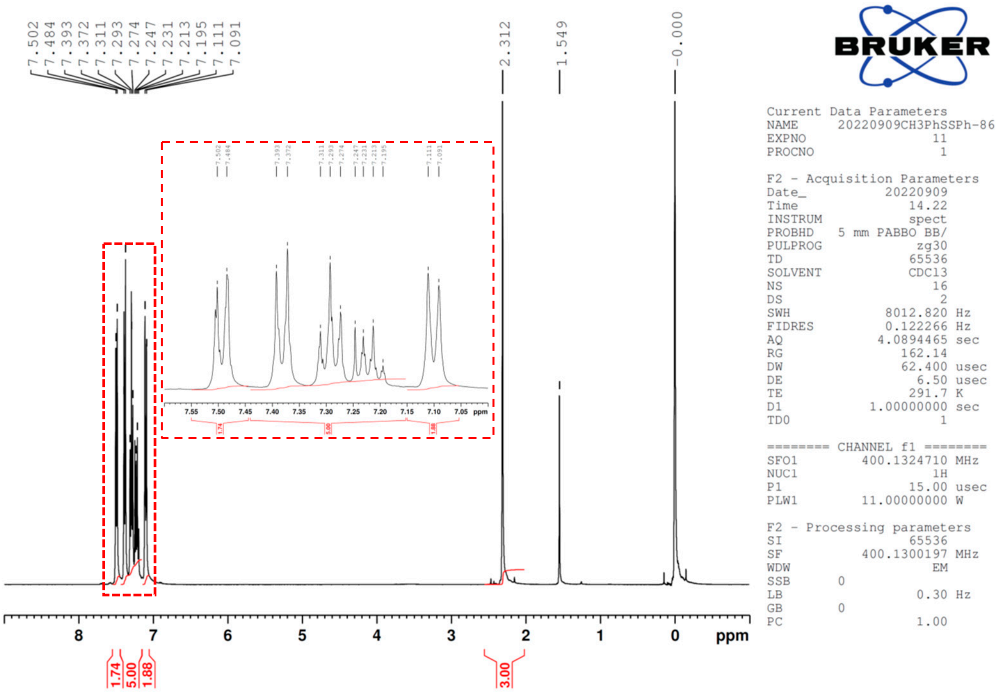

Figure S9. ^{1}H NMR spectrum of **2a**

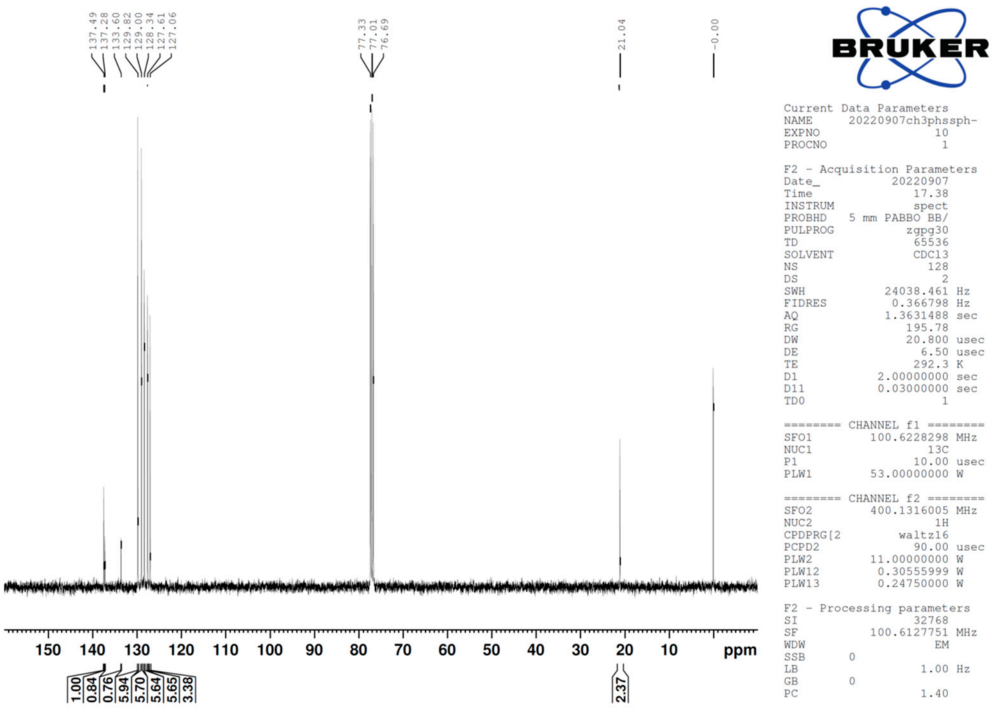

Figure S10. ^{13}C NMR spectrum of **2a**

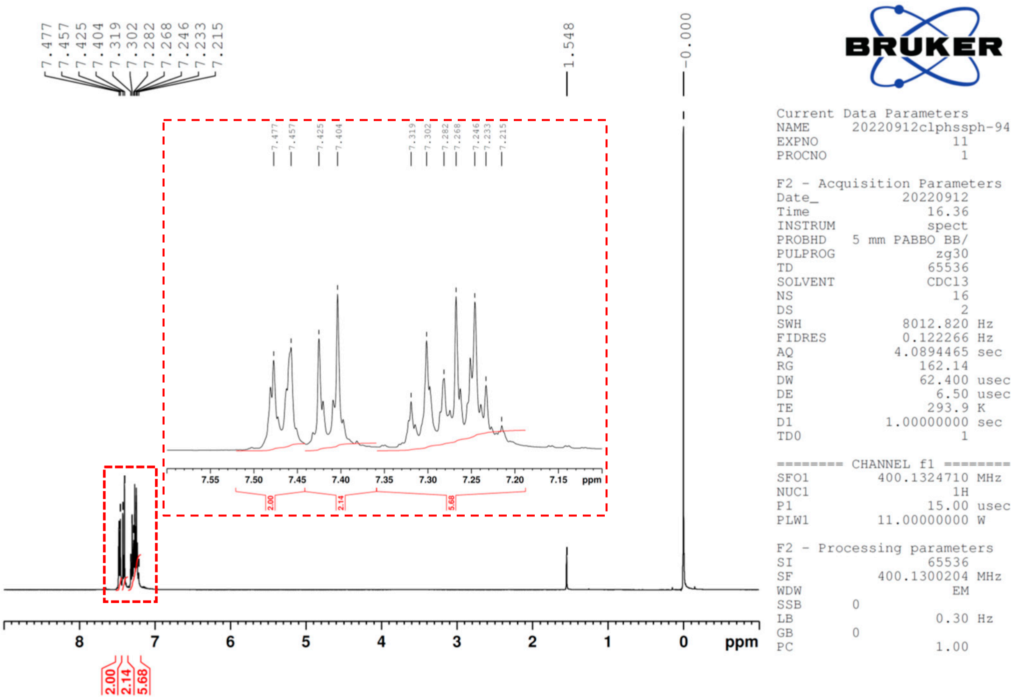

Figure S11. ^{1}H NMR spectrum of **2b**

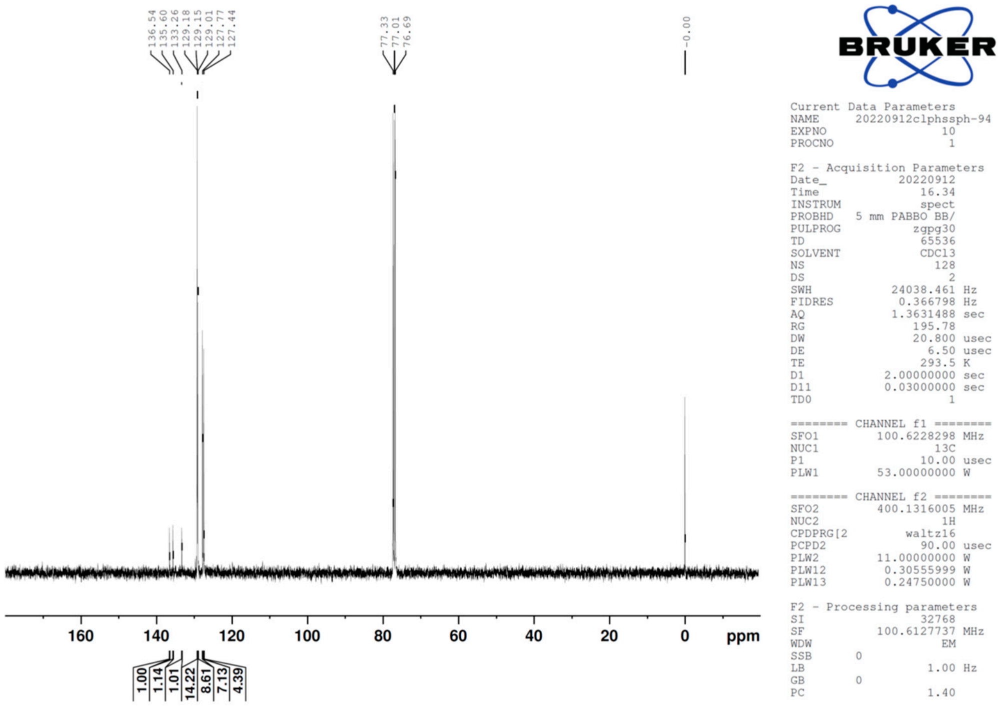

Figure S12. ^{13}C NMR spectrum of **2b**

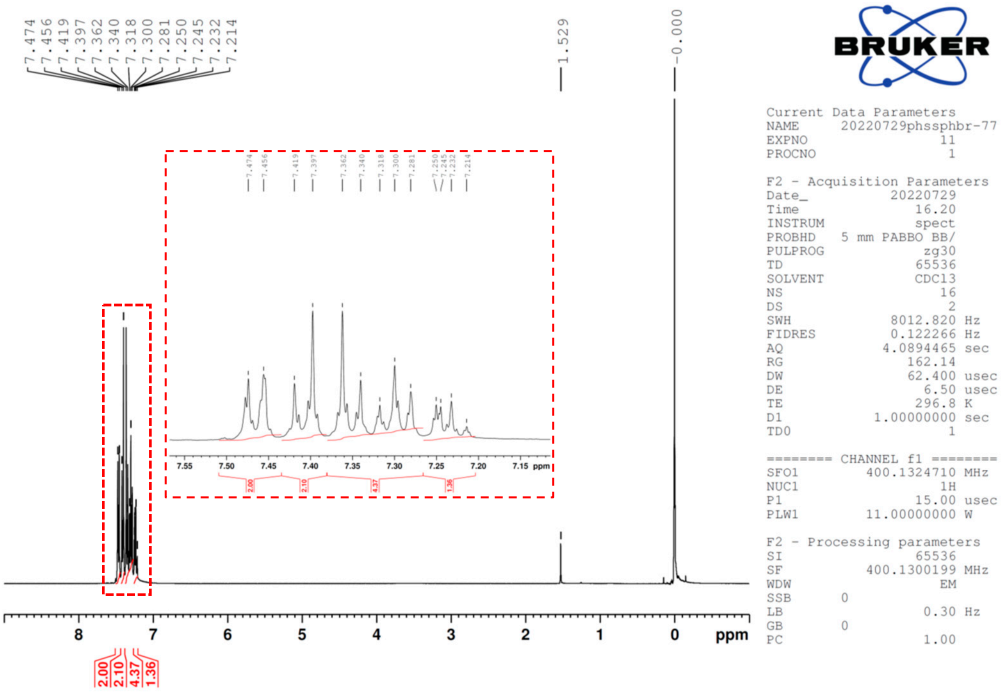

Figure S13. ^{1}H NMR spectrum of **2c**

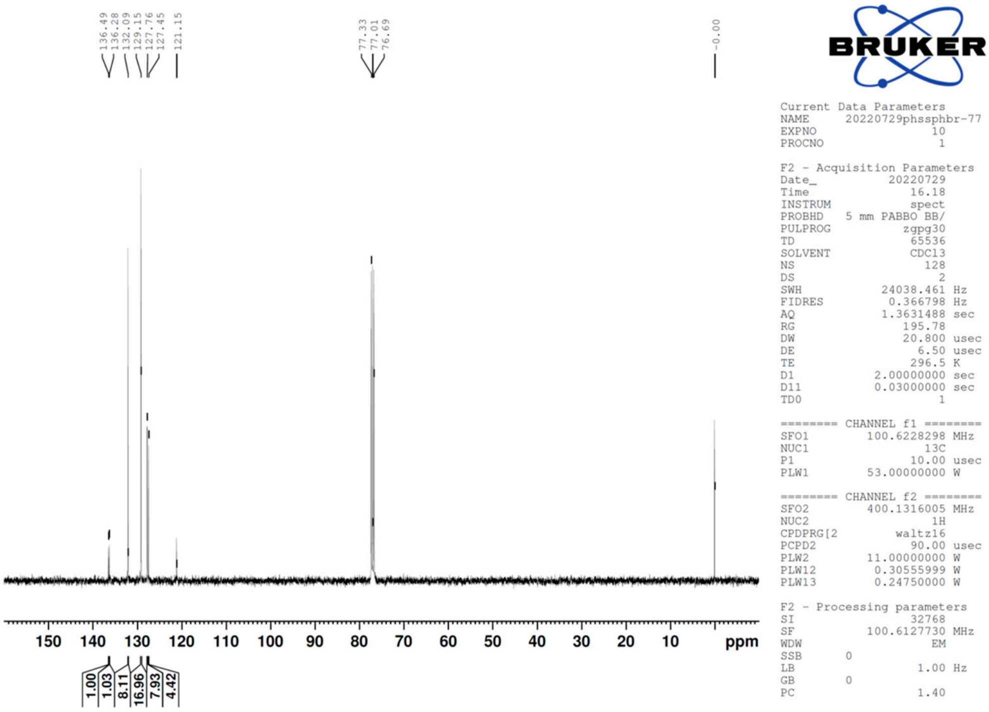

Figure S14. ^{13}C NMR spectrum of **2c**

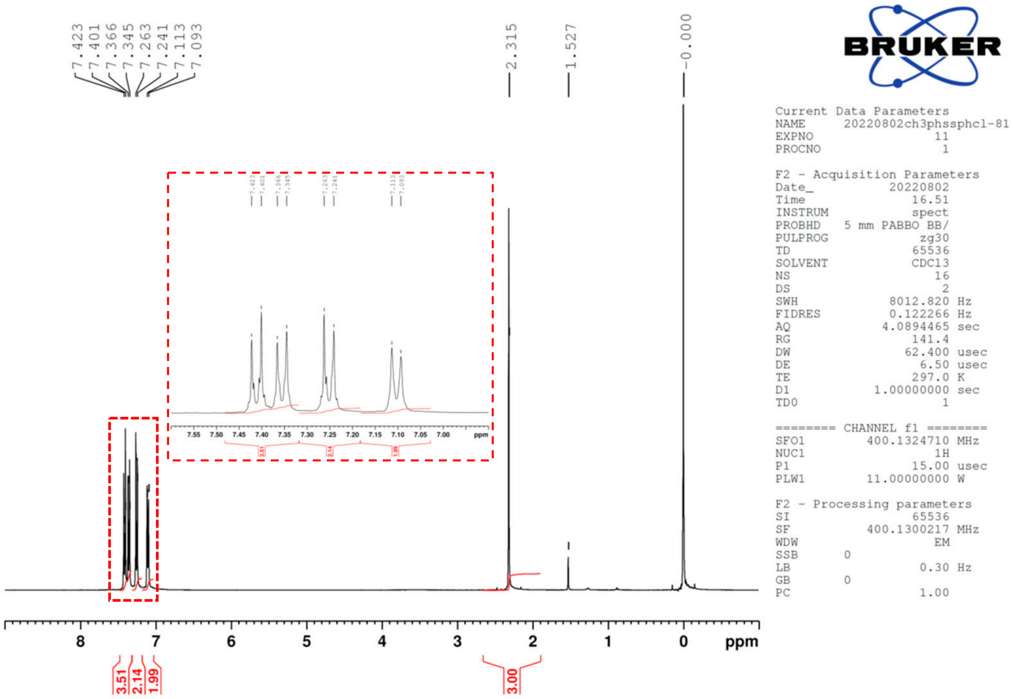

Figure S15. ^{1}H NMR spectrum of **2d**

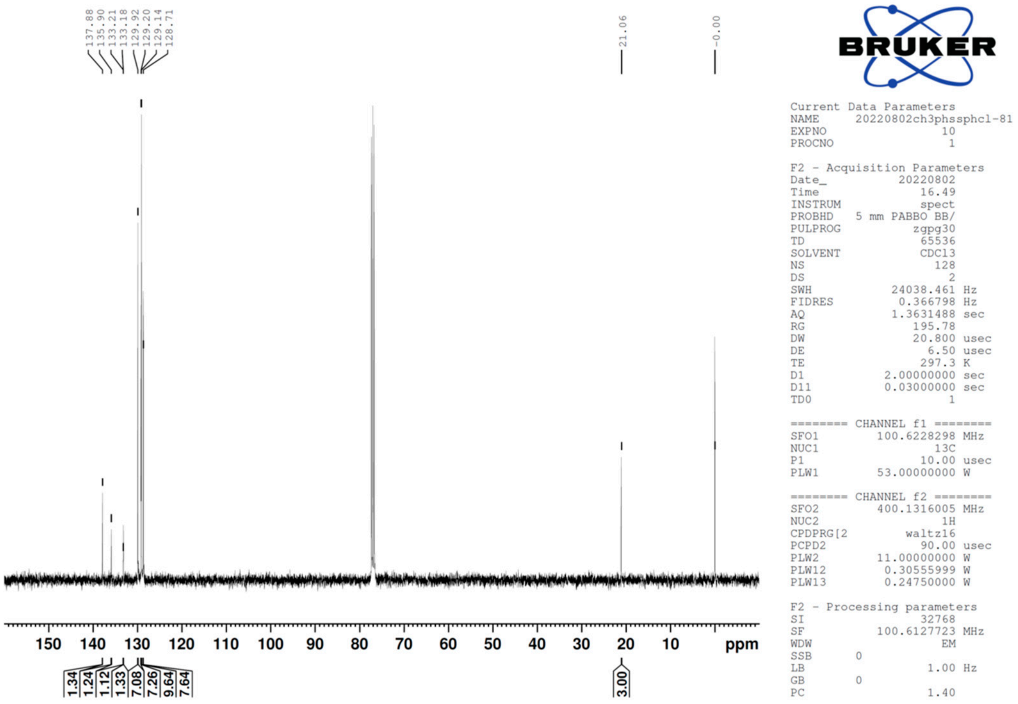

Figure S16. ^{13}C NMR spectrum of **2d**

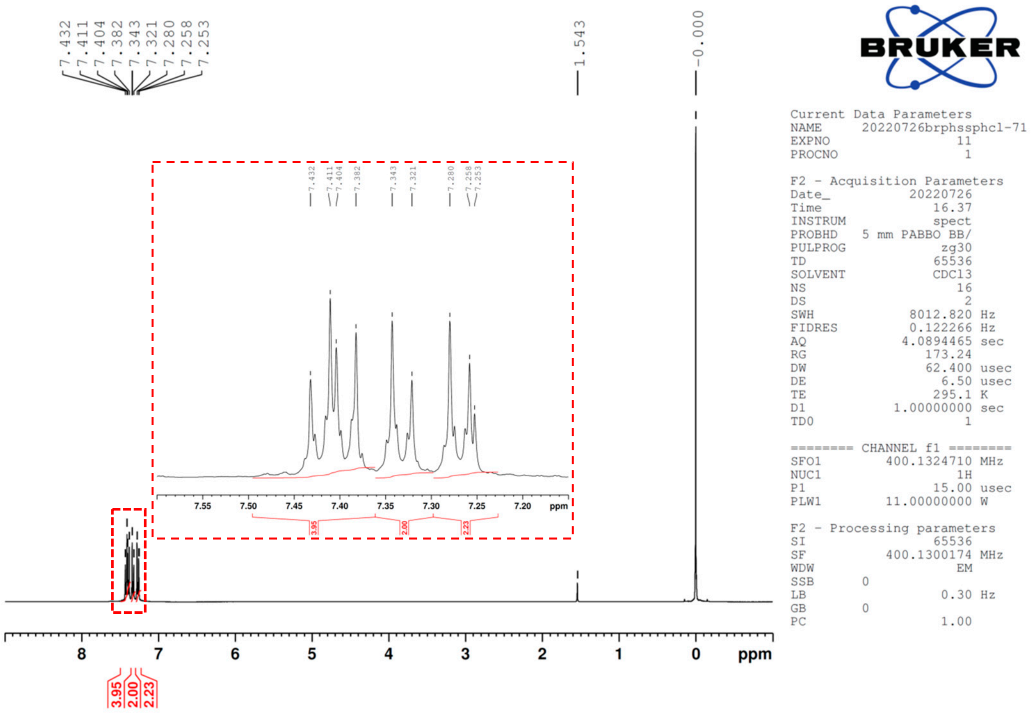

Figure S17. ^{1}H NMR spectrum of **2e**

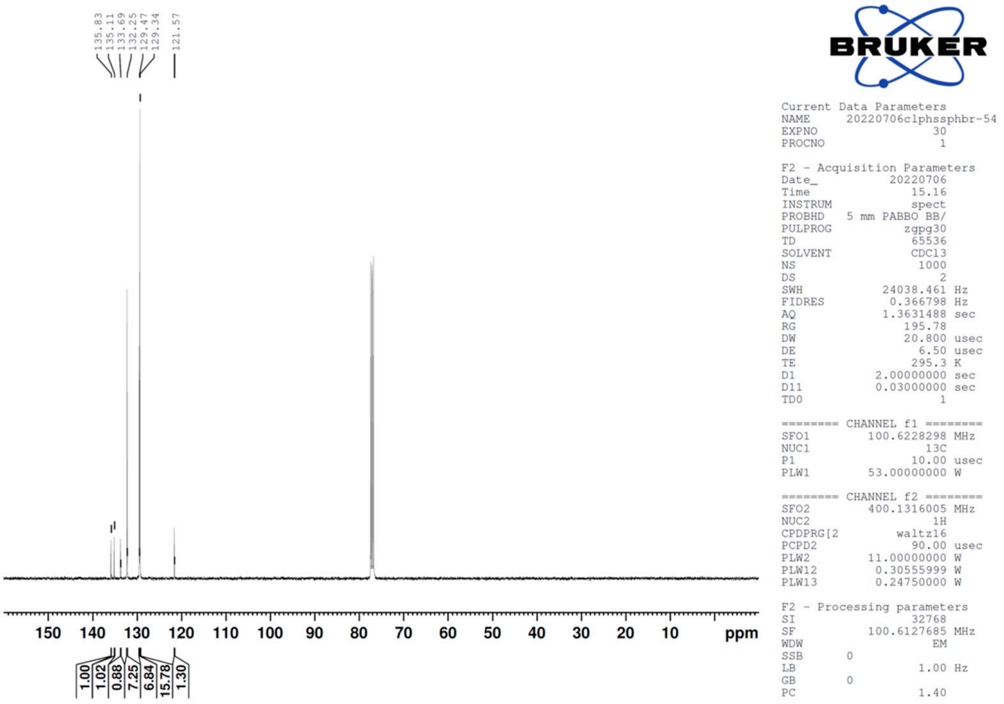

Figure S18. ^{13}C NMR spectrum of **2e**

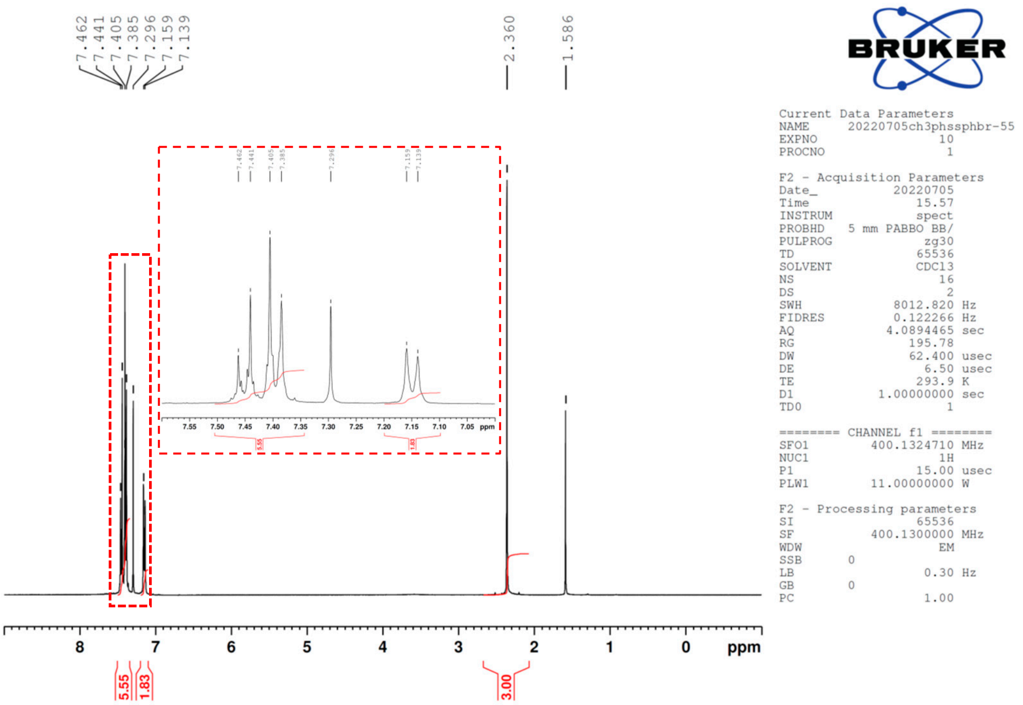

Figure S19. ^{1}H NMR spectrum of **2f**

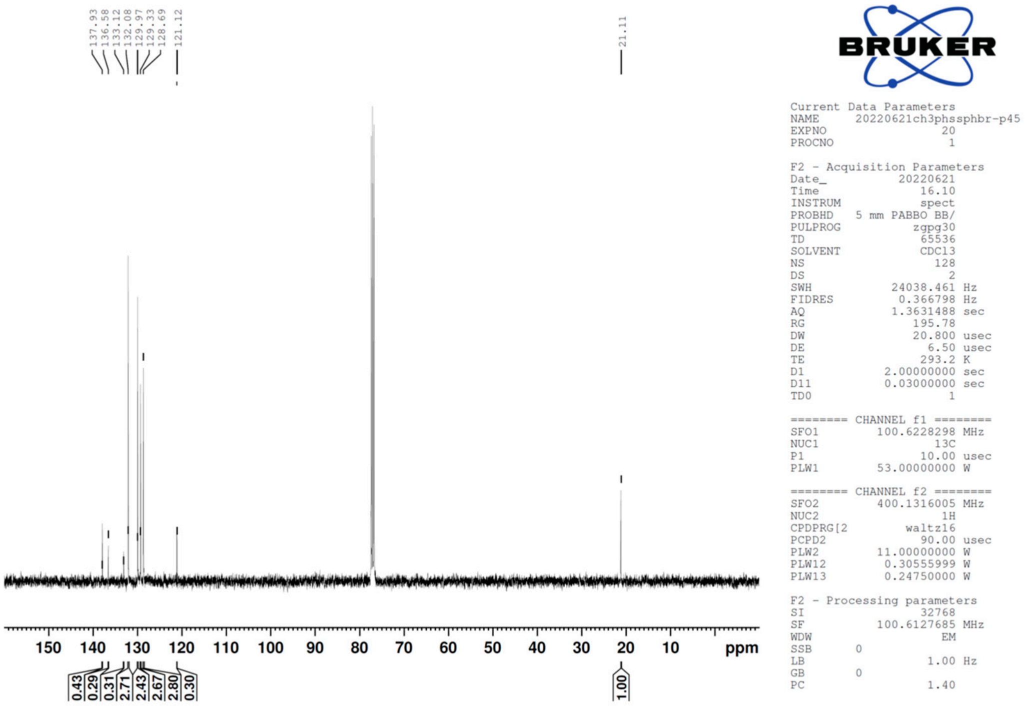

Figure S20. ^{13}C NMR spectrum of **2f**

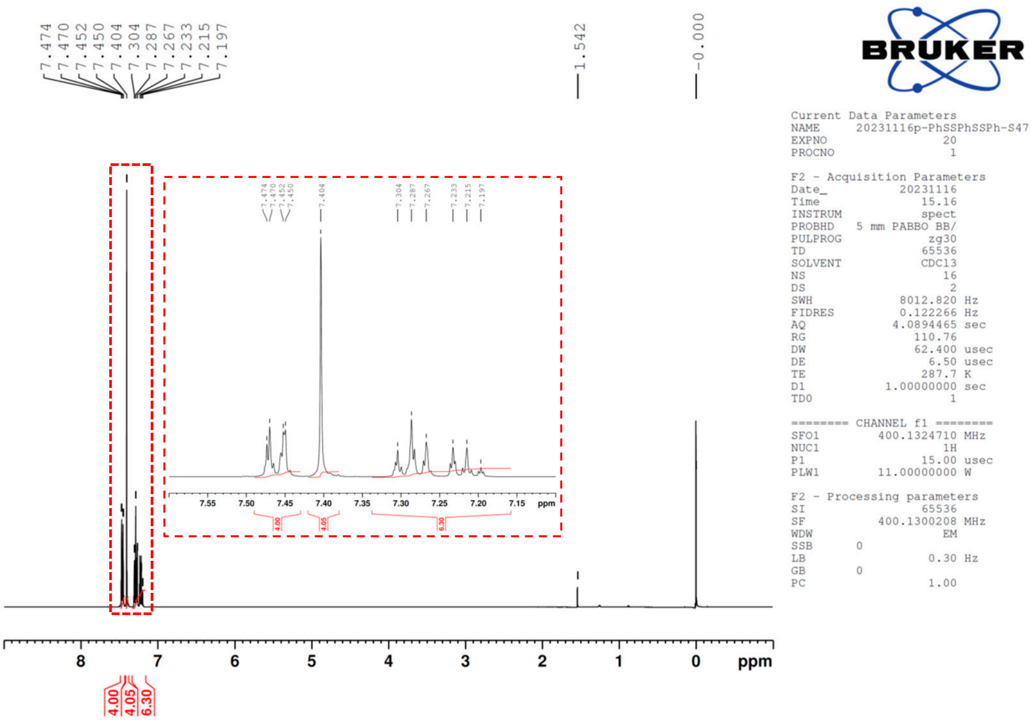

Figure S21. ^{1}H NMR spectrum of **2g**

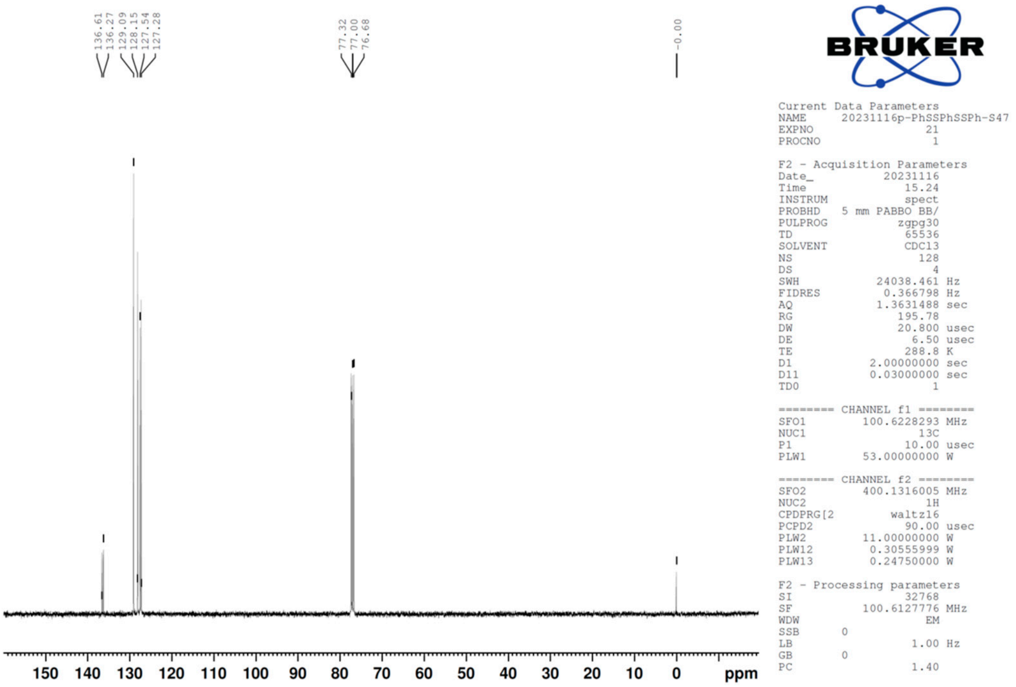

Figure S22. ^{13}C NMR spectrum of **2g**

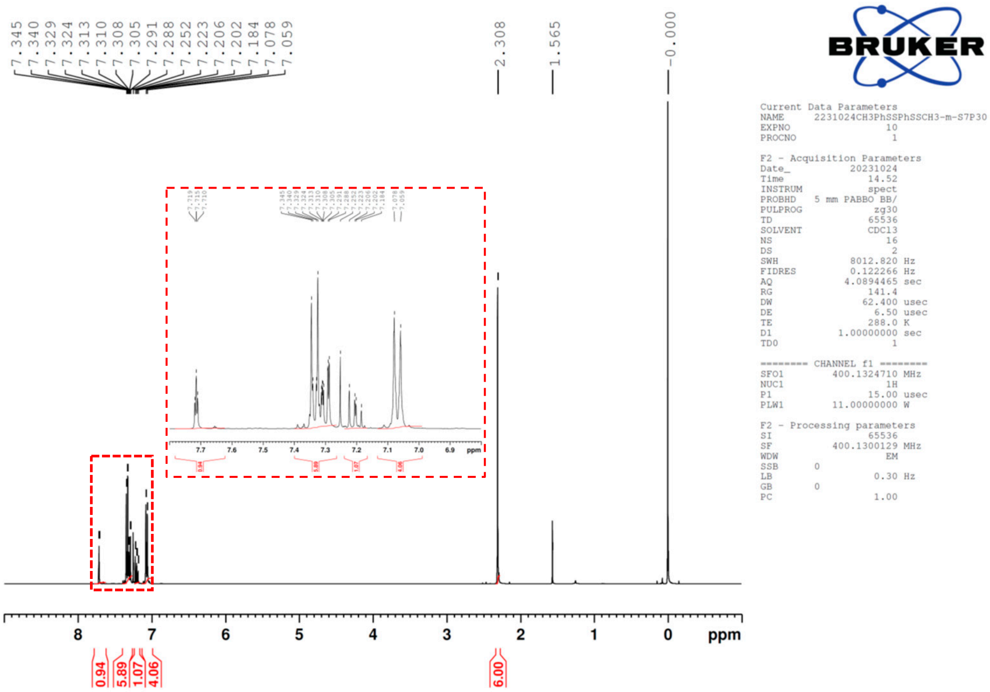

Figure S23. ^{1}H NMR spectrum of **2h**

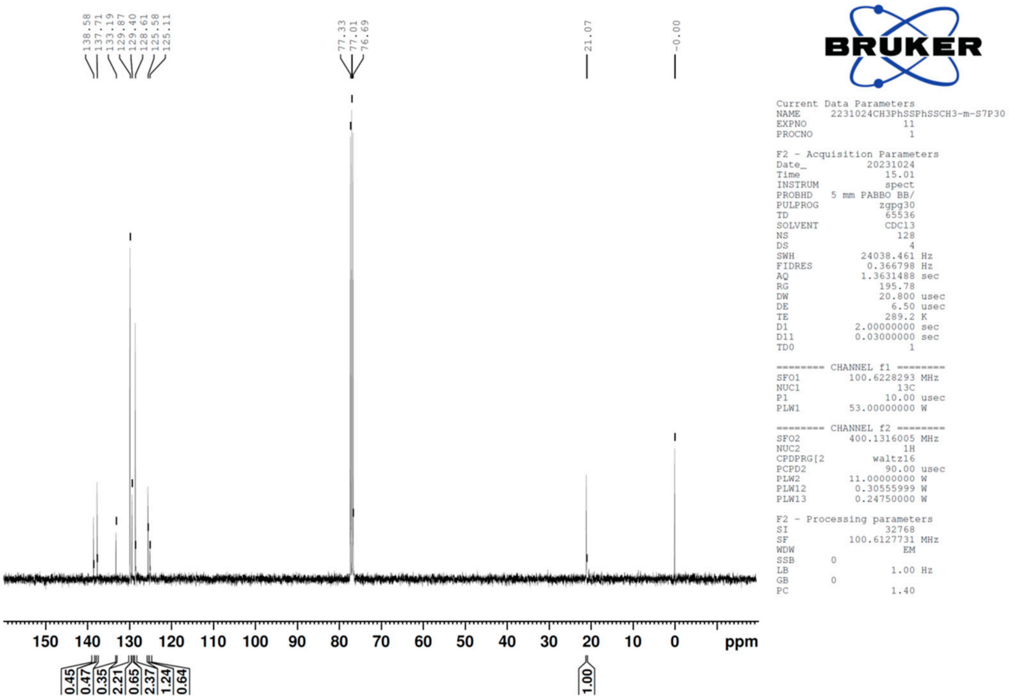

Figure S24. ^{13}C NMR spectrum of **2h**

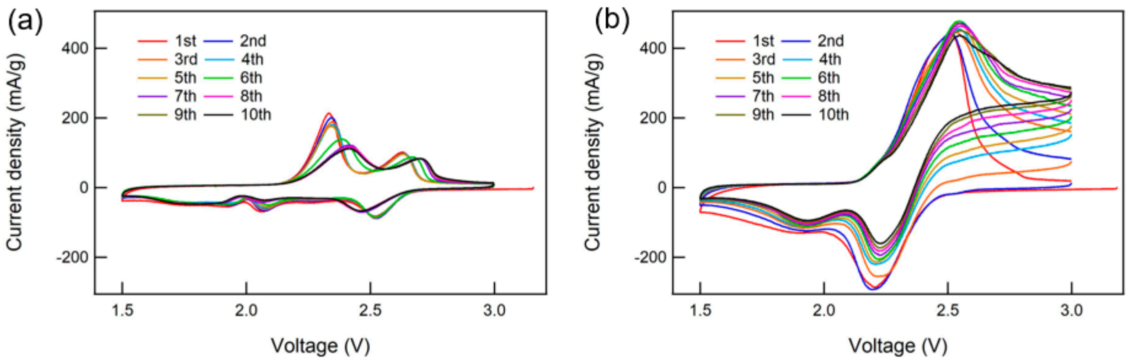

Figure S25. Cyclic voltammograms of **2g** (a) and **2h** (b)

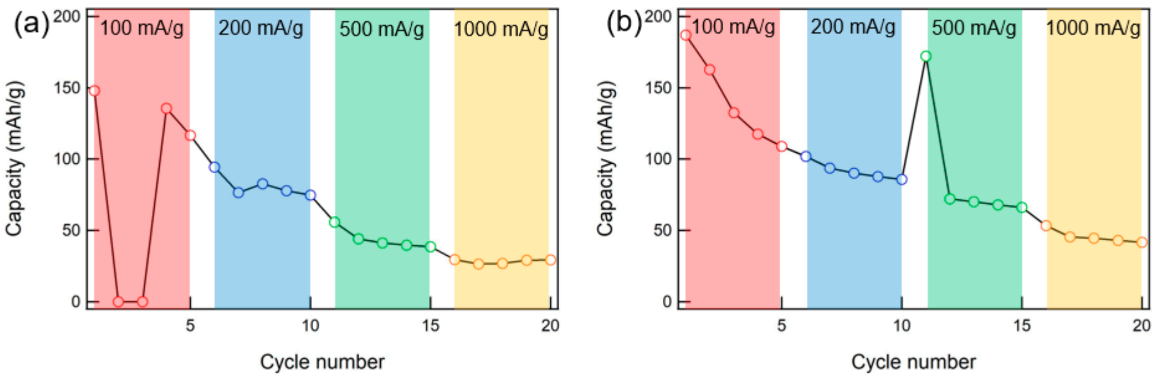

Figure S26. Rate performances of **2g** (a) and **2h** (b)

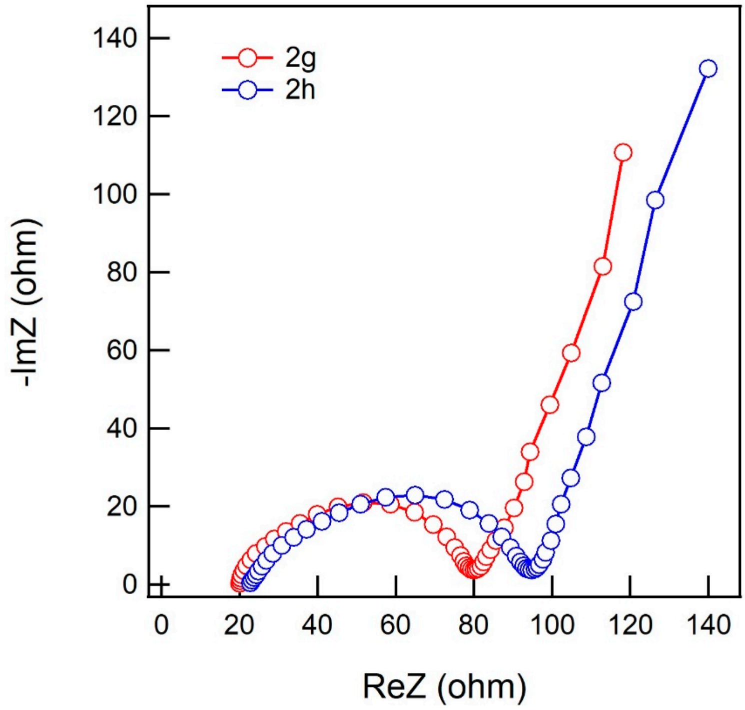

Figure S27. Nyquist plots of **2g** and **2h**

Table S1. Charge transfer resistances of organodisulfides

Organodisulfides	R_{ct} (ohm)	References
2, 2′-((disulfanediylbis (4,1-phenylene)) bis(azanediyl)) bis (naphthalene-1, 4-dione) (**MNQ**)	160	S1
4, 4″-dithiodianiline (**MDS**)	336	
2,3,4,6,8,9,10,12-Octathia biscyclopenta[b,c]-5,11-anthraquinone-1,7-dithione (**TPQD**)	150	S2
**2g**	60	This work
**2h**	72	

References

S1. Pei Yu, Jiaxuan An, Zhongju Wang, Yongzhu Fu, and Wei Guo, An Organic Molecular Cathode Composed of Naphthoquinones Bridged by Organodisulfide for Rechargeable Lithium Battery, *Small*, **2023**, 2308881.

S2. Zulipiya Shadike, Hung-Sui Lee, Chuanjin Tian, Ke Sun, Liang Song, Enyuan Hu, Iradwikanari Waluyo, Adrian Hunt, Sanjit Ghose, Yongfeng Hu, Jigang Zhou, Jian Wang, Paul Northrup, Seong-Min Bak, and Xiao-Qing Yang, Synthesis and Characterization of a Molecularly Designed High-Performance Organodisulfide as Cathode Material for Lithium Batteries, *Adv. Energy Mater.* **2019**, *9*, 1900705.
